# Macrobenthic community responses to multiple environmental stressors in a subtropical estuary

**DOI:** 10.7717/peerj.12427

**Published:** 2021-12-07

**Authors:** Fernanda M. Souza, Eliandro R. Gilbert, Kalina M. Brauko, Luciano Lorenzi, Eunice Machado, Mauricio G. Camargo

**Affiliations:** 1 University of Amapá State-UEAP, Macapá, AP, Brazil; 2 Centro de Estudos do Mar, Universidade Federal do Paraná, Pontal do Paraná, Paraná, Brazil; 3 Instituto Brasileiro de Geografia e Estatística-IBGE, Macapá, AP, Brazil; 4 Coordenadoria Especial de Oceanografia, Universidade Federal de Santa Catarina-UFSC, Florianópolis, SC, Brazil; 5 Department of Biology, University of Joinville Region-UNIVILLE, São Francisco do Sul, SC, Brazil; 6 Programa de Pós-Graduação em Saúde e Meio Ambiente, Institute of Oceanography, Federal University of Rio Grande-FURG, Rio Grande, RS, Brazil

**Keywords:** Macrofauna, Metal contamination, Estuarine gradient, AMBI, Organic enrichment, Babitonga Bay, Pollution effects, Sediments, Environmental health

## Abstract

We assessed how multi- and univariate models reflect marine environmental health based on macrobenthic community responses to three environmental stressor categories: hydrodynamics, organic enrichment and metal contamination. We then compared the models with the benthic index AMBI (AZTI Marine Biotic Index). Macrobenthic community and physicochemical variables were sampled at 35 sites along Babitonga Bay, a subtropical estuary in Southern Brazil. Distance-based linear modelling identified depth, grain size and organic matter as well as Cu and Zn as key stressors affecting the macrobenthos. Using canonical analysis of principal coordinates (CAP), we developed three multivariate models based on the variability in community composition, creating stress gradients. The metal gradient showed better correlation with the benthic community. Sediment quality indices (Geoaccumulation Index and Contamination Factor) showed a low to moderate contamination status, with higher concentrations for Cr, Ni and Zn at the inner areas of the bay. According to AMBI, Babitonga Bay has a “good” environmental health status, and the AMBI values show stronger correlations with the hydrodynamic and organic enrichment gradients (r = 0.50 and r = 0.47) rather than the metal gradient (r = 0.29). Lumbrineridae polychaetes (not included in the AMBI list) and *Scoloplos* sp. were negatively related to the metal contamination gradient and were considered sensitive, while *Sigambra* sp., *Magelona papillicornis*, the gastropod *Heleobia australis* and species of the crustacean order Mysida were positively related to the gradient and considered tolerant to higher concentrations of metals in the sediment. Despite the inconsistency in the ecological classification provided by AMBI and its relationship with the metal gradient, our results suggest that the environmental quality was satisfactory for the studied gradients. The metal gradient showed the weakest correlation to AMBI. In such cases, the ecological classification of taxa by the index should be evaluated under the perspective of the action of inorganic genotoxic contaminants represented by metals.

## Introduction

The structure of ecosystems is strongly modified by multiple environmental stressors that operate by cumulative and interactive mechanisms that are still largely unknown (Crain, Kroeker & Halpern, 2008; Sala et al., 2000; Alter et al., 2021). The intensity of anthropogenic impacts and the number of stressors has increased significantly in recent decades as a result of increasing demand for natural resources and increases in urban populations, especially along coastal environments (Halpern et al., 2007). Stressors are commonly associated with the introduction of various types of contaminants, loss of habitats, overexploitation of species (Jackson et al., 2001), introduction of invasive organisms and climate change (Sala et al., 2000; Kappel, 2005).

Estuaries are especially vulnerable ecosystems for receiving continental and urban discharge from extensive drainage basins, retaining sediments, nutrients, organic compounds, heavy metals and other contaminants. The ecological services provided by marine ecosystems range from contaminant retention, food production and recreation areas, to the generation of people’s cultural identity. The sustainability of their goods and services depends on highly complex ecological processes and the diversity of habitats within each ecosystem and are only possible due to the multitude of supporting and regulating services that underpin them (Thrush et al., 2013; Hope, Paterson & Thrush, 2020). Identifying the cumulative and interactive impacts is fundamental to evaluate the stress level of these ecosystems and to design more integrative and logical management strategies in real situations.

Marine environmental quality can be estimated through a series of univariate measures of different nature, such as indicator species, diversity measures and contaminant levels (Borja, Franco & Pérez, 2000; Rosenberg et al., 2004; Labrune et al., 2012). The ability to detect the impacts of univariate measures alone, however, allows limited interpretation in different habitats. This means that similar diversity values can be obtained for different species in two habitats (Clarke, 1993; Dufrene & Legendre, 1997). Multivariate biotic indices have therefore been widely adopted for coastal health assessments for expressing functional changes due to the most varied impact vectors. Multivariate models incorporate biological responses from the entire community, including both the number of species and the type of taxa associated with relative abundance or biomass measurements (Hewitt, Anderson & Thrush, 2005; Anderson, 2008). These functional indices can generate more sensitive and ecologically relevant diagnoses using the same effort required to generate univariate indices (Pohle, 2001; Hewitt, Anderson & Thrush, 2005; Hewitt et al., 2009). The structure of communities is usually determined through multivariate techniques, which have been successfully applied to detect the effects of pollution, including the differentiation of community responses when subjected to different degrees of disturbance (Ellis, Schneider & Thrush, 2000; Ellis et al., 2015; Hewitt, Ellis & Thrush, 2016).

Benthic macroinvertebrates are key elements in nutrient cycling and secondary production, as carbon sources that support higher trophic levels in many ecosystems. They act on nutrient flow processes between sediments and the water column through bioturbation and bioirrigation, with consistent responses to environmental changes, especially to physical and chemical stressors affecting the sediment (Quintino, Elliott & Rodrigues, 2006). For these reasons, benthic organisms are often used as environmental health indicators (Thrush et al., 2013; Chiarelli & Roccheri, 2014), assuming a fundamental role in decision-making that guides conservation strategies in real impact situations (McLusky & Elliott, 2004). However, the effect of multiple combined stressors with different degrees of impact on the benthic community can be non-linear (Hunsicker et al., 2016) and species’ responses are diverse and not clearly predictable (Crain, Kroeker & Halpern, 2008; Przeslawski, Byrne & Mellin, 2015).

Estuaries are the most studied marine ecosystem due to their intense anthropic exploration and essential services to coastal habitats, however, few studies have been conducted in the South Hemisphere. Therefore, a general goal is to enhance knowledge for more suitable and efficient uses of macrobenthic species as bioindicators. In this context, the main objective of this study was to examine if fauna assemblages are mostly affected by natural gradients (sediment distribution, depth, salinity, pH) or by anthropic contamination (organic enrichment, metal concentrations). To access that, we established three stressor categories *a priori* and investigated how univariate and multivariate models reflect the health of subtropical estuarine environments, based on macrobenthic community responses to variations in intensity and range area of multiple environmental stressors, knowingly: hydrodynamic, organic enrichment and metal contamination.

## Materials & methods

### Study area

**Figure 1:**
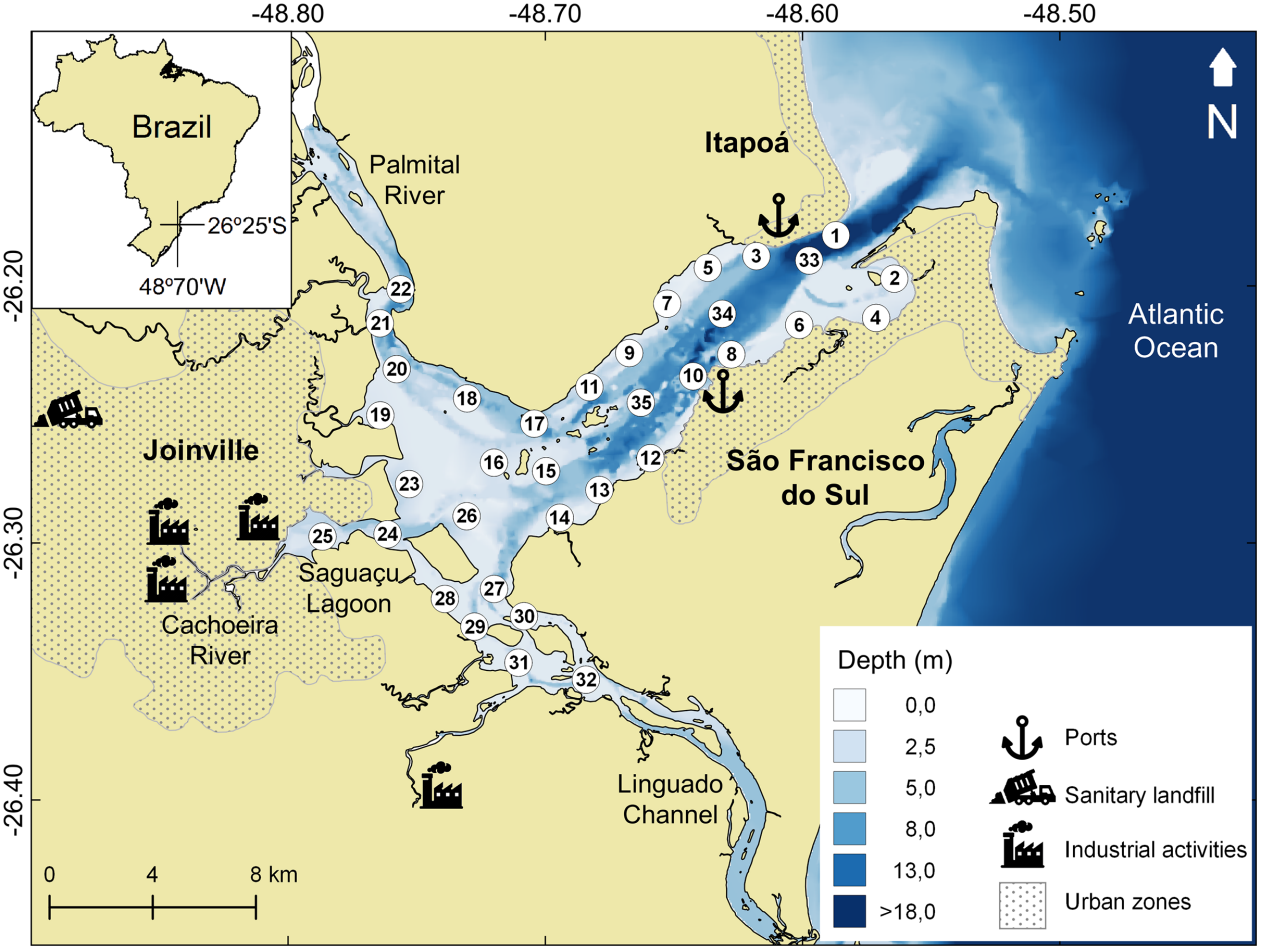
Babitonga Bay map and sampling sites. Study area indicating the 35 sampled sites, location of urban areas, industrial activities, landfill and São Francisco do Sul and Itapoá ports.

Babitonga Bay (26°15′S; 48°40′W) is one of the largest estuarine systems in the Southern Atlantic (160 km^2^) (Fig. 1). Its watershed drains an area of 1,400 km^2^, where agricultural activities such as fruit and grain production. The industrial sector develops metallurgical, mechanical and textile activities, as well as plastic, chemical, timber, mining and food industries (FATMA, 2002). The main channel connects the Atlantic Ocean to the Linguado channel and Palmital river, in the inner area (Noernberg, Rodrigo & Luersen, 2020), which also receives domestic and industrial effluent from adjacent cities (Cremer, 2006; Barros et al., 2010). In 2017, the domestic sewage collection benefited only 30% of the municipality’s population, with three treatment plants in operation (Sistema Nacional de Informações sobre Saneamento, 2019). The remaining domestic effluent and that from most economic activities was discharged in natura into the drainage network. The absence of proper wastewater treatment facilities is a reality, and sewage pollution is considered a problem for the Babitonga Bay ecosystem (Martins et al., 2014).

Before the Linguado Channel’s (SE axis) closure, in 1930s, the channel formed a connection to the sea that allowed greater circulation and water exchange in the internal region of the bay (Engel et al., 2017). Since then, the decreased circulation and water renewal inside the bay have generated a sedimentation and deposition area of fine sediments with the tendency to accumulate and potentiate the effects of the pollutants brought by the tributary rivers (FATMA, 2002).

Babitonga Bay has been historically contaminated by metals in its inner portion (Oliveira et al., 2006; Vaz et al., 2013), due to the discharge of untreated effluent from the municipalities and the industries nearby. Contamination by domestic effluents and linear alkylbenzenes (LABs) has also been documented near the main urban centers (Barros et al., 2010; Martins et al., 2014). The expansion of port activities along the estuary is also a relevant factor since dredging is necessary for the maintenance of navigation channels, causing the resuspension of contaminated sediments, making contaminants available to adjacent areas (Roberts, 2012; Silveira et al., 2012).

### Sampling and sample processing

The field surveys occurred in May 2014. Samples were taken using a stainless-steel Petersen bottom sampler (surface area of 0.0567 m²), at 35 sites along the estuary (Fig. 1), covering the largest possible area including regions close to potential sources of pollution. At each site, a sediment sample was taken for analysis of metals (chromium, nickel, copper, lead and zinc), macrofauna, total organic carbon, nitrogen, phosphorus, calcium carbonate and granulometry, as well as measurements of salinity, water temperature and the pH of the surface sediment.

For metal analysis, the stainless-steel Petersen bottom sampler was previously rinsed with Extran detergent, with a sampling area of 0.0567 m². Sediments were transferred to plastic trays and the surface sediment layer stored in acrylic vials. All samples were properly identified and kept on ice. In the laboratory, sediment samples were frozen (−20 °C). After completely frozen sediments were freeze-dried for 72 h, macerated on agate mortar, and sieved through a 63 µm nylon mesh to separate the cohesive fraction. Sieving reduces possible biases caused by the variability on sediment grain size (Birch, 2003; Salomons & Förstner, 1984). Samples were then partially extracted through microwave assisted acid digestion (Milestone Ethos Plus), following the EPA 3051A (US EPA, 2007) protocol. This method extracts the more easily mobilized metals from the surface sediments, which presents the highest potential of exposition and assimilation by local fauna (Luoma, Ho & Bryan, 1995).

After digestion samples were transferred to decontaminated falcon tubes, filtered to eliminate the insoluble fraction, and diluted to 50 ml with a 2% HNO3 solution. Extracts were kept refrigerated until determination of the elements chromium (Cr), copper (Cu), nickel (Ni), lead (Pb), and zinc (Zn) through inductively coupled plasma-optical emission spectrometry (ICP-OES) in an Optima 7000 DV ICP-OES (PerkinElmer, Waltham, MA, USA). Average recoveries of Standard Reference Material BCR701-Lake Sediment (Extractable Trace Elements), Method Quantification Limit (MQL) and Method Detection Limit (MDL) (Table S1).

Sediment grain size was determined through laser diffraction using a MICROTRAC Bluewave. Granulometric parameters were calculated using the method of moments in R (R Core Team, 2020) using the rysgran package (Gilbert, Camargo & Sandrini, 2012). Total organic matter (TOM) concentrations were determined using the gravimetric method after combustion in a furnace at 550 °C for 1 h. Total organic carbon (TOC) and nitrogen (TN) were determined by a dry combustion method with a Perkin Elmer 2400 CHN analyzer. Total phosphorus was determined using the colorimetric method described by Grasshoff, Erhardt & Kremling (1983). Calcium carbonate (CaCO_3_) was determined by the weight difference before and after sample digestion by a hydrochloric acid (HCl) solution at 1 mol·L^−1^.

For the macrofauna, three replicates were sampled at each site (total of 105 samples), placed in plastic bags and then fixed in 6% formalin (ICMBIO permit N 42056-1). After fixation, the samples were washed in a sieve with a 0.5-mm aperture and preserved in 70% alcohol. The retained material was sorted with the aid of a stereomicroscope, and the organisms were finally counted and identified to the lowest possible taxonomic level.

### Data analysis

We used univariate measures such as total abundance (N), mean value of the three replicates, number of species (S) and Shannon–Wiener (H’) diversity index. In addition, we calculated AMBI (AZTI Marine Biotic Index; Borja, Franco & Pérez, 2000) using the software available at http://ambi.azti.es. AMBI has been proved as a relatively reliable index to access environmental quality in Southern Brazilian estuaries (Brauko et al., 2015; Brauko et al., 2016; Checon et al., 2018) and it is based on the distribution of species abundance into five ecological groups, ranked according to their sensitivity to a progressive gradient of stress (EG I–sensitive, EG II–indifferent, EG III–tolerant, IV second-order opportunistic and EG V–first-order opportunistic) as displayed in the model of Grall & Glémarec (1997). AMBI results vary from 0 (high environmental quality) to seven (extremely polluted environment) (Borja, Franco & Pérez, 2000, 2003). The classification of AMBI values was based on the work of Muxika, Borja & Bonne (2005): “high”, <1.2; “good”, 1.2–3.3; “moderate”, 3.3–4.3; “poor”, 4.3–5.5; and “bad”, >5.5.

To assess the level of contamination status at each site, we used the geoaccumulation index (*I*_*geo*_) and contamination factor (*CF*) for the selected metals: Cu, Cr, Ni and Zn. *I*_*geo*_ is a widely used index to assess the magnitude of the contamination of an individual element, based on the relation between the measured metal concentration and its reference value, with both being normalized in terms of the granulometry of the sediment sample using only the fine fraction of the sample (Müller, 1986). Contamination factor is a simple and effective tool to monitor heavy metal contamination, providing a sediment quality indicator to assess the pollution degree of sediments. *CF*_*metals*_ is the ratio between the metal concentration of the sample and the background values in the sediment, *CF*_*metals*_ *= C*_*metal*_*/C*_*background*_ (Hakanson, 1980).

$IGeo = \; {\log _2}\left[ {\displaystyle{{{C_n}} \over {1.5\; {B_n}}}} \right]$

The background values applied to calculate *I*_*geo*_ and the *CF* were from Kim et al. (2018).

We established three groups of variables *a priori*, here classified as environmental stressors, to verify if fauna distribution is mostly affected by natural estuarine gradients or environmental disturbance. The three environmental stressors groups were: hydrodynamic (depth, salinity, pH, grain size, sorting, CaCO_3_ and mud); organic enrichment (organic matter, total phosphorus, total nitrogen, total organic carbon); metal contamination (Ni, Cr, Cu, Pb, Zn). Then, we proceeded to investigate how univariate and multivariate models reflect the estuarine health status, based on macrobenthic community responses to variations in intensity and range area of multiple environmental stressors.

Distance-based linear models (DistLM) were applied to each category of stressor in order to select the variables that explained the highest percentage of variation in the benthic community. We used the variables corresponding to each gradient as predictor variables, and the Bray–Curtis dissimilarity matrix based on the main macrobenthic species representing more than 70% of the total abundance as the response variable. Species with high abundance and low occurrences (less than 10% of the replicates) were removed. A step-wise selection method was used, and the criterion for selecting the best model was the Akaike’s Information Criteria corrected for small samples (AICc). The DistLM analysis pointed out that for the hydrodynamic gradient, depth and average grain size were the most important variables. For the organic enrichment gradient, only organic matter (OM) was selected, and for the metal contamination gradient, Cu and Zn were selected. However, Cr and Ni were added to the model for presenting relatively higher concentrations in the samples analyzed (Table 1).

**Table 1:** Distance-based multivariate multiple regression analysis (DistLM) between stressor variables and macrobenthic fauna showing the selected variables for the three stressor gradients.

	Variable	Pseudo-F	*P*	%
Hydrodynamic	Salinity	2,705	0.002	7.58
	Depth	5,431	0.000	14.13
	pH	3,622	0.000	9.89
	CaCO_3_	1,919	0.030	5.50
	Mud	2,492	0.004	7.02
	Grain size	4,235	0.000	11.37
	Sorting	1,313	0.203	3.83
	Best solution: depth, grain size (23.00%)			
Organic enrichment	TP	2,544	0.003	7.16
	TOC	2,157	0.012	6.14
	TN	1,658	0.072	4.78
	OM	2,717	0.001	7.61
	Best solution: OM (7.6%)			
Metal contamination	Cr	2,607	0.003	7.32
	Ni	2,422	0.005	6.84
	Cu	2,207	0.011	6.27
	Pb	2,243	0.009	6.36
	Zn	3,352	0.000	9.22
	Best solution: Cr, Ni, Cu, Zn (24.17%)			

**Note:**

Variables considered were: Hydrodynamic (Salinity, Depth, pH, CaCO_3_, Mud, Grain Size, Sorting); Organic Enrichment (TP, Total Phosphorus; TOC, Total Organic Carbon; TN, Total Nitrogen; OM, Organic Matter); Metal Contamination (Cr, Chromium; Ni, Nickel; Cu, Copper; Pb, Lead; Zn, Zinc). Selection criterion: AICc. Selection procedure: Step-wise. DF: 33.

To reduce multidimensional information to a single variable representing the environmental stress gradient, the variables selected by the DistLM for each category were submitted to a principal component analysis (PCA), and the scores of the first main component were extracted. For the hydrodynamic gradient (depth and grain size), the PCA axis 1 explained 67.7% of the variation and for the metal contamination gradient (Cu, Zn, Cr and Ni), the variation explained by axis 1 was 88%. A non-hierarchical clustering analysis was then performed on each gradient to identify possible groups of samples and to generate “ecological categories”, which ranged from “healthy” to “impacted”, as a way of assessing the ecological quality of an environment. The values of the environmental variables for each category of each gradient were then calculated and are shown in Table 2. For each environmental gradient, the differences between the ecological categories were tested with PERMANOVA and pair-wise comparisons. All categories were significatively different (*P* < 0.05). It is important to consider that this classification between healthy and impacted refers only to the sampling sites of this study and should not be used as a parameter of comparison in other locations. For instance, the Babitonga Bay sites considered to be heavily impacted by metals in this study may not be classified as highly impacted on a global scale.

**Table 2:** Ecological Categories (EC) for the three multivariate models: hydrodynamic; organic enrichment and metal contamination.

	EC	n	Depth	Grain size (µm)			N	S	H’	AMBI
**Hydrodynamic**	1	2	**6.6** (5.9–7.3)	**802** (750–854)			**9.8** (5.7–14)	**4.5** (2.8–6.3)	**1.1** (0.8–1.5)	**1.28** (1.25–1.31)
	2	3	**18.6** (12.1–24.2)	**348** (321–384)			**27.6** (13.7–45)	**10.1** (6.3–15)	**1.8** (1.7–2.2)	**1.32** (0.76–1.82)
	3	5	**11.3** (5.6–16.4)	**240** (187–308)			**87.7** (3.7–210)	**12.4** (3–20.3)	**1.7** (0.8–2.1)	**2.19** (0.58–3.36)
	4	5	**6.5** (3.7–9.7)	**195** (71–301)			**87.6** (32–146)	**17.9** (11.7–27.3)	**2.1** (1.7–2.6)	**1.59** (0.78–2.99)
	5	6	**3.3** (2.6–3.7)	**146** (112–189)			**76.6** (25–297)	**12.1** (7.7–15.3)	**1.7** (0.6–2.4)	**2.53** (1.59–3.35)
	6	14	**1.5** (0.5–2.6)	**163** (77–223)			**139** (46–334)	**19.7** (8–39)	**2.1** (0.9–2.8)	**2.66** (1.47–4.12)
**Organic enrichment**	**EC**	**n**	**OM** **(%)**				**N**	**S**	**H’**	**AMBI**
	1	7	**0.98** (0.21–1.84)				**101** (5.7–334)	**16** (2.7–39)	**1.8** (0.8–2.8)	**1.45** (1.02–1.88)
	2	16	**3.6** (2.34–4.89)				**101** (3.7–231)	**16.4** (3–27.3)	**2** (0.8–2.6)	**2.15** (0.58–4.12)
	3	5	**6.59** (5.40–7.66)				**76** (25.3–165)	**16.3** (12.7–21)	**2.3** (2–2.6)	**2.54** (1.59–3.43)
	4	7	**10.5** (9.18–13.3)				**97** (25–297)	**11.7** (7.7–17.7)	**1.5** (0.6–2.4)	**2.93** (2.27–3.42)
**Metal contamination**	**EC**	**n**	**Cr** **(mg.kg** ^**−1**^ **)**	**Cu** **(mg.kg** ^**−1**^ **)**	**Ni** **(mg.kg** ^**−1**^ **)**	**Zn** **(mg.kg** ^**−1**^ **)**	**N**	**S**	**H’**	**AMBI**
	1	6	**35.3** (28.1–42)	**12.5** (10.7–15.3)	**13.5** (10.6–15.8)	**79** (46–103)	**85** (5.7–179)	**15.4** (2.7–25.7)	**1.9** (0.8–2.8)	**2.13** (1.25–4.12)
	2	9	**49.9** (42.2–58.2)	**15.7** (14.1–18.1)	**18.9** (15.9–20.6)	**112** (81–122)	**85** (15–334)	**17.4** (7.3–39)	**2.1** (1.7–2.6)	**2.15** (0.78–3.36)
	3	17	**65.2** (54.4–83)	**19.8** (17.3–23.8)	**24.2** (21.3–28.8)	**146** (113–188)	**111** (3.7–297)	**15.1** (3–24)	**1.9** (0.6–2.6)	**2.1** (0.58–3.81)
	4	3	**95.8** (87.7–102.2)	**37** (33.4–41.8)	**33** (26.6–41.9)	**288** (199–369)	**74** (26.3–144)	**10.9** (7.7–12.7)	**1.6** (1.5–2)	**3.3** (3.14–3.42)

**Note:**

Mean (bold), minimum and maximum values for the variables associated to each model and its categories. n, number of sites within each ecological category; Depth at sampling sites (m); Grain size, mean grain size; OM, Organic matter; Cr, Chromium; Cu, Cupper; Ni, Nickel; Zn, Zinc; N, species bundance per sample; S, taxa number per sample; H’, Shannon-Wiener index; e AMBI.

**Figure 2:**
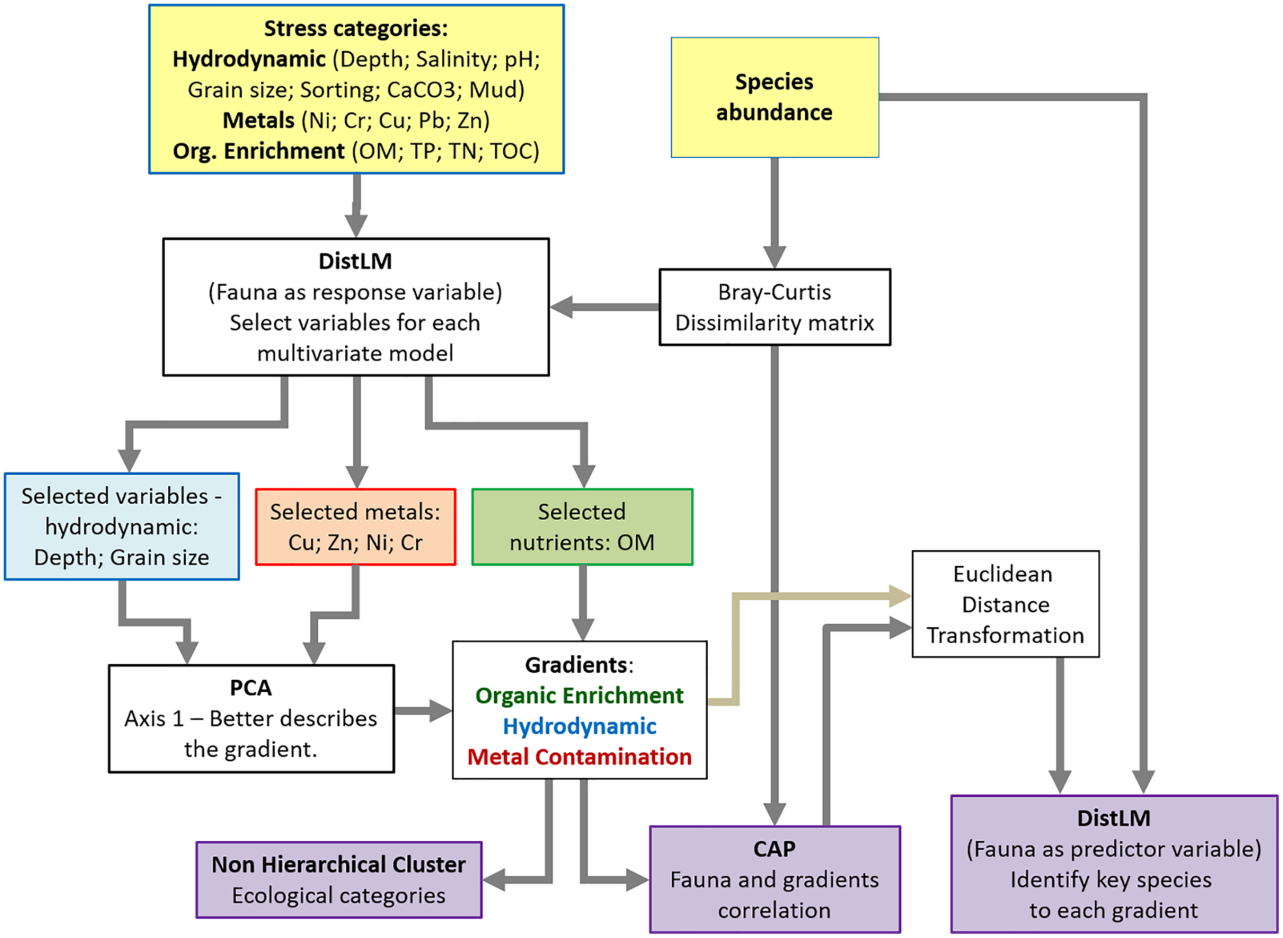
Flowchart of the statistical analysis.

In order to determine whether there was a significant relationship between the distribution and composition of macrobenthic communities and the three environmental stressor gradients, canonical analyses of principal coordinates (CAP) were performed. In CAP, the Bray–Curtis dissimilarity matrix created with the abundance data for the main macrofaunal species was confronted with each environmental stress gradient (generated by the PCA scores), resulting in the axis that best represented the macrofauna distribution in relation to each environmental stress gradient. Key, sensitive or tolerant, species, which were determinant for separation of the stress categories in each environmental gradient, were identified by DistLM using the abundance matrix as predictor variable and the CAP results as the response variable (Tables S2–S4). A flowchart of the analytical procedures can be seen at Fig. 2.

DistLM was also used to partition the variance explained by the three environmental gradients and determine how the gradients might interact to affect benthic community composition (Table S5). Relative percentage of benthic community variation explained by each stressor gradient were then calculated. PCA, PERMANOVA, DistLM, and CAP were performed using the PERMANOVA+ add-on package for PRIMER v6 (Clarke & Gorley, 2006; Anderson, Gorley & Clarke, 2008). All the other analyses and graphs were produced in R (R Core Team, 2020).

## Results

**Figure 3:**
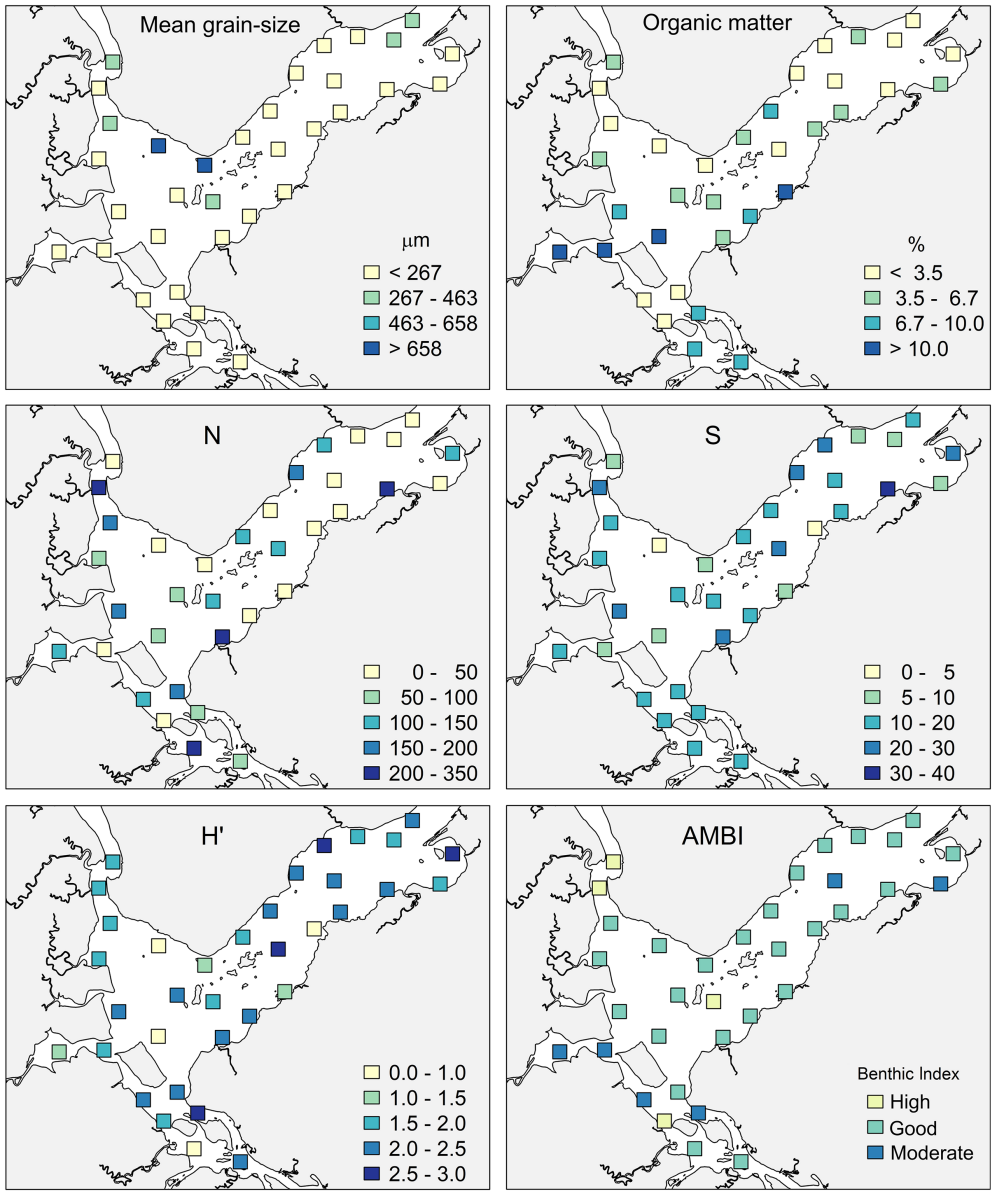
Mean grain size, organic matter percentage, macrofaunal total abundance (N), species number (S), Shannon–Wiener index (H’) and AMBI status at the 35 sampling sites in Babitonga Bay. Fine and Very Fine Sand (<267 µm); medium sand (267–463 µm), medium and coarse sand (463–658 µm), Coarse sand (>658 µm).

The sediments of Babitonga Bay are mostly fine poorly-sorted sand (227 ± 160 µm). The deepest sites are composed of medium and coarse sand, while the innermost sites (in Saguaçu lagoon) of very fine sand, with higher mud content (Fig. 3).

**Figure 4:**
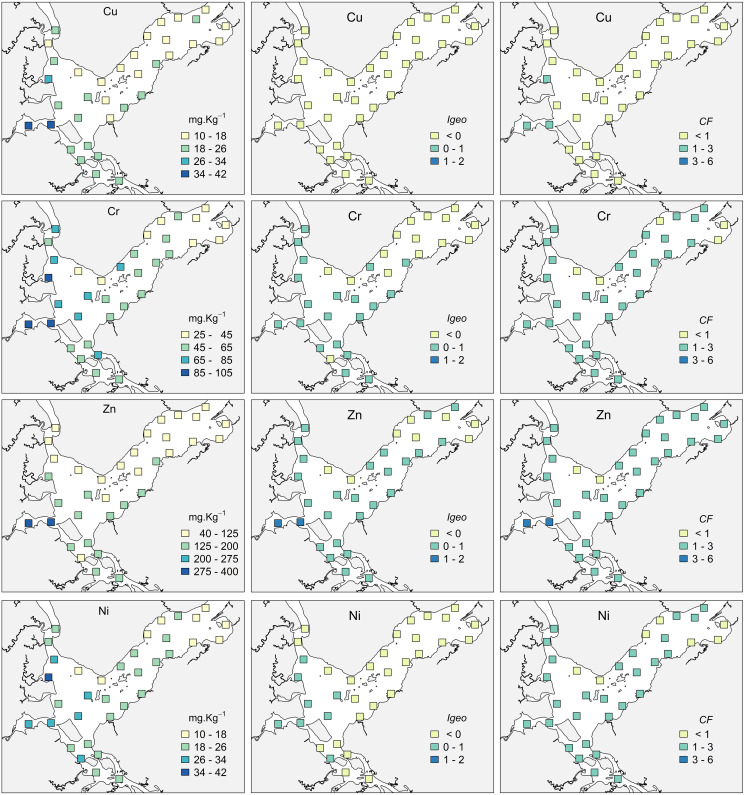
Concentrations, Geoaccumulation index (*Igeo*) and Contamination Factor (*CF*) for Cupper (Cu), Zinc (Zn), Chromium (Cr) and Nickel (Ni) at the 35 sampling sites in Babitonga Bay.

The highest concentrations of OM occurred in the internal areas of the estuary and in the Linguado channel (Fig. 3). These areas receive drainage from urban locations and have shallower depths and hydrodynamics, which favors the deposition of mud and OM. The metals had a similar distribution, with higher values at sites 24, 25 and 19, which are subjected to the urban drainage from Joinville city and, consequently, its industrial activities. The metals Cu and Zn presented maximum values in Saguaçu lagoon (41.83 for Cu and 369 mg.kg- for Zn), while Ni and Cr presented higher values at site 19 (41.86 and 102.18 mg.kg^−1^ respectively), located at the mouth of an artificial drainage channel also from Joinville city (Fig. 4).

Results of the *Igeo* and *CF* indices indicated that the majority of the Babitonga Bay presents a non-contaminated to moderately contaminated status (Fig. 4, Table S6). For Cu, *Igeo* indicated absence of contamination at all sites, and three sites with a moderate *CF*. For Zn, 25 out of 35 sites where assigned class 2 for *Igeo* (absent to moderately contaminated) and two sites were considered moderately contaminated (sites 24 and 25). According to *CF* results, 31 sites were considered with a moderate contamination factor for Zn and sites 24 and 25 with a considerable contamination factor. Cr and Ni had a similar classification, with most sites assigned as ‘absent to moderately contaminated’ for *Igeo* and with a moderate *CF*.

In total, 10.148 macroinvertebrate individuals belonging to 135 taxa were identified. Annelida had the highest abundance (60.5%) and Mollusca were sub-dominant (21.7%), followed by Arthropoda (12.8%), accounting for 95% of total abundance (Table 3).

**Table 3:** Total abundance, dominance and accumulated dominance of the main macrobenthic groups sampled at the Babitonga Bay.

Phylum	Abundance	%	Cumulative %
Annelida	6,142	60.52	60.52
Mollusca	2,201	21.69	82.21
Arthropoda	1,296	12.77	94.98
Nemertea	189	1.86	96.85
Cnidaria	107	1.05	97.90
Echinodermata	85	0.84	98.74
Platyhelminthes	56	0.55	99.29
Sipuncula	44	0.43	99.73
Chordata	25	0.25	99.98
Porifera	3	0.03	100
Total	10,148		

In the main channel of the estuary, the total abundance (N), number of species (S) and H’ index were higher near the margins and islands, while in the inner portion, the lowest values were recorded inside the Saguaçu lagoon and at the most internal site at the Palmital river (Fig. 3). Forty percent of the sites showed abundance lower than 50 individuals, while only four sites concentrated an abundance of more than 200 organisms, always close to the mouth of smaller streams.

The ecological status shown by AMBI was in general very homogeneous, with only four sites classified as “high”, six as “moderate” and 25 as “good”, most of them located in the main channel (Fig. 3). The internal region of the estuary showed more heterogeneous environmental qualities, with two sites classified as “high” status at the Palmital river, and sites attributed “moderate” status at Saguaçu lagoon and the Linguado channel. “Moderate” status also occurred at two sites in the outer area. No “poor” or “bad” statuses were diagnosed in the estuary.

**Figure 5:**
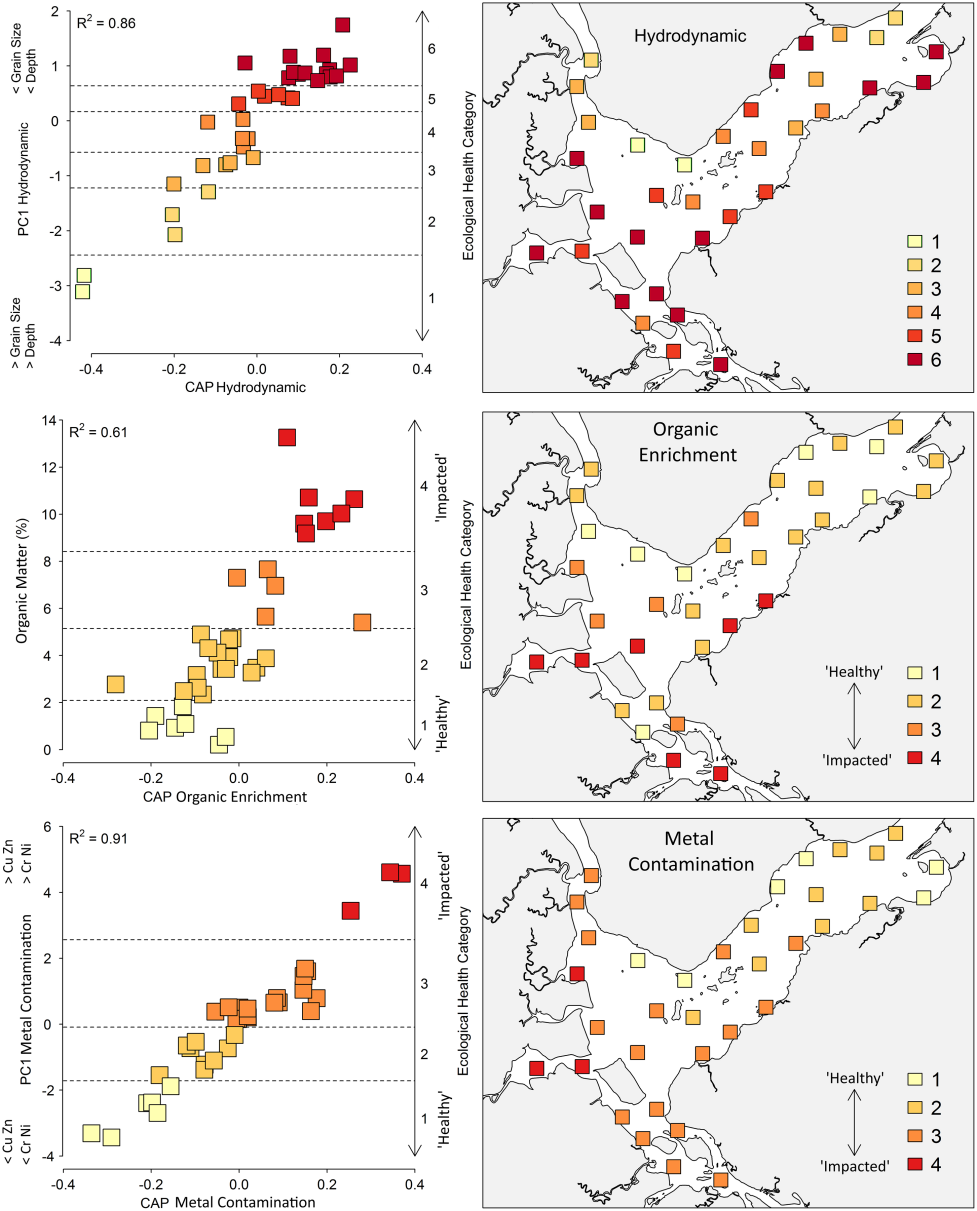
Canonical analyses of principal coordinates (CAP) and spatial distribution of the ecological categories based on the Hydrodynamic, Organic Enrichment and Metal Contamination multivariate model. For the Hydrodynamic model, horizontal lines indicate ecological categories ranging from one (higher depth and coarse sediments) to six (shallow areas with fine sediments). For the Organic Enrichment and Metal Contamination models, horizontal lines indicate ecological categories ranging from one (‘Healthy’) to four (‘Impacted’).

There was a strong correlation between the benthic community and the hydrodynamic gradient, which includes the variables mean grain size and depth (R^2^ = 0.86) (Fig. 5). The hydrodynamic gradient was divided into six categories, ranging from coarser sediments (750 to 854 μm) at greater depths (5.87 to 7.31 m) in category 1, to a mean diameter of 77 to 223 μm along with depths from 0.52 to 2.55 m in category 6 (Table 2).

Category 6 accounted for 40% of the 35 sites, with shallower depths and smaller sediments. These sites are mostly located in the shallow, less turbulent areas of the bay, Saguaçu lagoon, the Linguado channel and the southern region, which favors the deposition of fine sediments (Fig. 5). Categories 1–3 incorporated the sites of the mouth of the bay and deeper areas within the Palmital river, which receive large continental water inputs. Abundance, number of species, Shannon–Wiener diversity and AMBI values were lower in category 1, and progressively increased towards category 6 (Table 2). The correlation between AMBI and hydrodynamic gradient was relatively high (r = 0.50) when compared to that with other gradients, organic enrichment (r = 0.47), and metal contamination (r = 0.29).

Nemerteans, crustaceans of the Mysida order, polychaetes of the Capitellidae family and the gastropod *Bulla striata* were negatively related to the hydrodynamic gradient, showing affinity for shallower areas and for finer sediments, while polychaetes from the Syllidae family were positively related (Table 4) (DistLM results are shown at Supplementary Material). AMBI classified the taxon positively related to the gradient with affinity for deeper areas and larger sediments in ecological group II (indifferent to stress). On the other hand, the negatively related species were classified in groups II–IV, representing indifferent, tolerant and second-order opportunistic.

**Table 4:** Key species of each Ecological Gradient selected by Distance-based linear model (DistLM).

Gradient	Response	Taxon	Family	Class	EG
**Hydrodynamic**	−	Nemertea			III
	−	Mysida		Malacostraca	II
	−	Capitellidae	Capitellidae	Polychaeta	IV
	−	*Bulla striata*	Bullidae	Gastropoda	II
	**+**	Syllidae	Syllidae	Polychaeta	II
**Organic**	**+**	Mysida		Malacostraca	II
**Enrichment**	−	*Scoloplos* sp.	Orbiniidae	Polychaeta	I
	**+**	*Sigambra* sp.	Pilargidae	Polychaeta	III
**Metal Contamination**	**+**	*Sigambra* sp.	Pilargidae	Polychaeta	III
	−	Lumbrineridae	Lumbrineridae	Polychaeta	–
	**+**	*Heleobia australis*	Cochliopidae	Gastropoda	IV
	**+**	Mysida		Malacostraca	II
	−	*Scoloplos* sp.	Orbiniidae	Polychaeta	I
	**+**	*Magelona papillicornis*	Magelonidae	Polychaeta	I

**Note:**

Taxa that presented a negative response were considered sensitive. Taxa that presented a positive response were considered tolerant to the stressors of each gradient. Family, Class and AMBI Ecological Group (EG).

The organic enrichment gradient was characterized only by the percentage of OM and was divided into four categories. Macrofaunal assemblages were strongly related to OM (R^2^ = 0.61), with 45% of the sites classified into category 2 (2.34% to 4.89% OM). Category 1 (0.21% to 1.84% OM) grouped sites at the Palmital river and the main channel of the bay, while category 4 (9.18% to 13.26% OM) grouped the sites at the Saguaçu river, Linguado channel and proximities (Fig. 5). The abundance of organisms and number of species were higher within the sites of category 1, progressively decreasing towards category 4. AMBI values agreed with this gradient (r = 0.47), with lower values for category 1 sites and higher values for category 4 sites (Table 2).

The polychaete *Scoloplos* sp. was negatively related to the organic enrichment gradient, in opposition to species from the crustacean order Mysida and the polychaete *Sigambra* sp., which were positively related, showing affinity for higher concentrations of OM (Table 4). AMBI classified the species inversely related to the gradient as sensitive (EG I), while the species classified into EGs II and III, or indifferent and tolerant, agreed with the gradient.

The metal contamination gradient was determined by Cu, Zn, Cr and Ni concentrations. This gradient showed the strongest correlation between changes in the benthic community and sediment metals (R^2^ = 0.91), indicating that this model is highly suited for determining the effects of increasing metal concentrations on fauna (Fig. 5). All metals increased from category 1 to 4, and 48% of the sites were allocated into category 3, comprising the entire internal and median regions of the estuary (Table 2). Sites 19, 24 and 25, which directly receive the urban drainage from Joinville, were in category 4 (impacted), and were also classified into the highest categories of the hydrodynamic and organic enrichment gradients. They also displayed the lowest values for abundance and number of species, an indication of the higher impacts in Babitonga Bay.

The polychaete family Lumbrineridae (not included in the AMBI list) and the genus *Scoloplos* sp. (EG I) were negatively related to the metal contamination gradient and were considered sensitive, while *Sigambra* sp. (EG III), *Magelona papillicornis* (EG I), the gastropod *Heleobia australis* (EG IV) and the crustacean order Mysida (EG II) were positively related to the gradient and considered tolerant to higher concentrations of metals in the sediment (Table 4). In this gradient, *M. papillicornis* was inconsistent with the ecological classification of AMBI, showing an incoherent pattern of affinity. The other taxa that were positively related to the metal gradient were classified either as indifferent, tolerant, or as second order opportunists by AMBI, while the genus negatively related to the gradient, considered sensitive by the model, is also classified as sensitive by the index. The correlation between AMBI and the metal contamination gradient itself was also weak, the lowest among the three gradients analyzed (r = 0.29).

Variance partitioning showed that the differences in hydrodynamics and in metals individually explained the higher proportions of variation in the faunal composition (Table 5). Most of the variance was explained by the interaction among the three gradients (43.34%) in relation to the isolated factors and may be a result of metal ion affinity with the fine mud sediment fraction, making less hydrodynamic areas more susceptible to deposition of metals, OM and other contaminants.

**Table 5:** Relative percentage of benthic community variation explained by different stressor gradients.

Stressor gradient	%
Hydrodynamic	23.00
Organic enrichment	7.60
Metal contamination	24.17
Hydrodynamic * Organic	28.93
Hydrodynamic * Metal	38.35
Organic * Metal	28.34
Hydrodynamic * Organic * Metal	43.34

**Note:**

Calculated by variance partitioning method.

## Discussion

According to our results, the majority of Babitonga Bay can be considered as minimally impacted, except for the innermost areas. The most impacted sites were in the internal region, comprising the Saguaçu lagoon, Linguado channel, and its southern region, that receives direct drainage from Joinville and São Francisco do Sul cities. These are shallow areas, with extensive intertidal flats and mangroves with higher potential for deposition and accumulation of fine sediments and contaminants (Bonatti et al., 2004; Martins et al., 2014, Noernberg, Rodrigo & Luersen, 2020). For Cu, Ni and Zn, the majority of the sites presented a moderate *CF*, while *Igeo* presented a similar result to Cr and Zn only. The element with the higher *CF* and *Igeo* were Zn, at the Saguaçu lagoon, where the concentrations for this element were higher. When comparing the Babitonga metal levels with other Brazilian estuaries, our levels were higher than found in Paranaguá bay (Angeli et al., 2020), although Paranaguá bay data are from the whole sediment while our data were obtained from the fine fraction only. Therefore, our results are more comparable with Baixada Santista, a highly urbanized and industrialized estuary (Kim et al., 2018), with similar Cr (14.43–103.82 mg.kg^−1^) and Cu (5.5–112.29 mg.kg^−1^) concentrations, higher Ni (5.39–19.7 mg.kg^−1^) and lower Zn (44.74–983.90 mg.kg^−1^) concentrations.

CAP shows that the three environmental gradients indicated by our analysis were well correlated with macrofaunal variation patterns, denoting their relevance in dynamics and distribution of soft-bottom organisms. Among the multiple environmental stressors, the benthic macrofauna distribution was more affected by sediment metal concentration. Nevertheless, it is also important to consider that most changes in the macrofaunal assemblages were explained by the interaction among the three gradients. This was expected, since the fate of metals in estuarine environments is closely linked to hydrodynamics and local sedimentation processes. Metals have been associated with the presence of suspended particulate matter and also the presence of iron and manganese oxyhydroxides in the sediments, usually linked to the fine-grained fraction (Burton, 2002; Yamamuro & Kanai, 2005).

According to the environmental quality index AMBI, the bay generally has a “good” ecological status, which was expressed for 71% of the sites, mostly located in the main channel. However, when comparing the AMBI ecological classification with our environmental quality gradients, there was better agreement only for the hydrodynamic and organic enrichment gradients (r = 0.50 and r = 0.47), while its correlation with the metal contamination gradient was much weaker (r = 0.29). Only to the sites at Saguaçu lagoon, which are in a more impacted category, were given a “moderate” status.

Faunal responses to organic matter concentrations are essentially a response to changes in nutrient pathways, feeding modes and physiological tolerance mechanisms to the decreasing oxygen availability associated with this type of disturbance (Souza et al., 2013; Bon et al., 2021). Consequently, organically enriched environments tend to be dominated by more generalist and opportunistic taxa or r-strategist guilds, which are physiologically more resistant to oxygen deprivation (Pearson & Rosenberg, 1978; Cardell, Sardà & Romero, 1999; Brauko et al., 2016). Such faunal responses are the basis of AMBI’s function, which was originally designed as an organic enrichment model (Borja, Franco & Pérez, 2000). In addition, the index has performed adequately when applied to polycyclic aromatic hydrocarbons and other oil-derived impacts (Muniz et al., 2005; Muxika, Borja & Bonne, 2005), which indeed represent organic sources of contamination and trigger responses by means of nutritional enrichment of benthic fauna. AMBI has also showed positive correlations to various contamination proxies in a bay in Southeastern Brazil; however, its correlation was lower for heavy metals (Checon et al., 2018).

The mechanisms by which metal contaminants damage organisms can be largely distinct from the mechanisms involved in organic pollution. One of the pathways most affected by metals is genotoxicity, which involves the breakage and loss of nuclear genetic material in somatic cells, and eventually gametes (Amiard-Triquet, Mouneyrac & Berthet, 2013; Nunes et al., 2017). This type of mechanism triggered by metals at sub-cellular levels will be manifested in the higher biological levels of populations or communities only in the next generations or sub-populations of the species impacted, after longer periods of exposure (Morgan, Kille & Stürzenbaum, 2007; Borja et al., 2011). Our results suggest that AMBI might be less sensitive to this kind of stress, especially to possible short-term effects at sub-cellular levels unless they are already reflected at the community level, after the settlement of local new generations.

In this sense, species classified by AMBI as sensitive to excessive OM may perform in the opposite way under the influence of metals. We did find inconsistencies between one key sensitive species determining the separation of the stress categories in the metal environmental gradient, *Magelona papillicornis*, and its ecological group determined by AMBI (EG I). The remaining key taxa for the metal gradient and also for the hydrodynamic and organic enrichment gradients, where all coherent to their EGs within the AMBI list. The crustacean order Mysida was a key taxon for the three gradients analyzed, occurring in finer sediments and less hydrodynamic areas with higher organic enrichment and metal contamination levels. Mysid shrimps are commonly used as standard species in metal toxicity studies (Verslycke et al., 2004) to measure various sublethal toxicant effects, such as growth, swimming capability, feeding behavior, reproduction, sexual maturity, and vitellogenesis. Estuarine mysids have a flexible physiology that responds to the changing environmental variables in the complex chemistry of estuaries. However, the available evidence suggests that mysids are generally more sensitive to toxic substances than many other test species (Roast et al., 1998; Hunt et al., 2002; Verslycke et al., 2003) and are suitable for monitoring through behavioral toxicity tests for predicting the influence of pollutants (Roast, Widdows & Jones, 2000). Thus, the metal concentration in Babitonga Bay sediments may not be high enough for a populational response but, since mysids are sensitive to metal contaminants, it is likely that sublethal effect may occur, and weren’t detected by biological indices.

Ambiguities in diagnostics provided by AMBI have been pointed out under multiple contamination stressors including metals and organic enrichment (Cai et al., 2014; Rabaoui et al., 2015; Tweedley, Warwick & Potter, 2015; Brauko et al., 2015, 2016), symptoms of the need for further studies involving manipulative field experiments and multivariate approaches. The reality in this case is especially captured by means of multivariate approaches, where the selected models incorporated the variables affecting or governing the faunal structure despite the co-occurrence of multiple stressors with synergistic effects, at different intensities and impact scales.

Regarding environmental quality, our results indicate that Babitonga Bay is, in general, under a moderate degree of impact, mainly in the internal areas with weak hydrodynamics and higher sedimentation rates, factors that favor the accumulation of contaminants associated with suspended particulate matter. Although hydrodynamic characteristics and OM content are important factors affecting communities, the structure of benthic assemblages were more significantly correlated with the metal contamination gradient. The changes in community structure indicate possible toxic effects, with the potential to be transferred to higher levels of the estuarine trophic chain. Once incorporated by benthic fauna, metals can bioaccumulated and be transferred to higher trophic levels, posing a risk to human consumption (Doğan-Sağlamtimur & Kumbur, 2010; Casado-Martinez, Smith & Rainbow, 2013; Kalman et al., 2014). These areas are more vulnerable and should be included in monitoring programs by the environmental agencies responsible for local management.

Efforts to control the disposal of effluents, such as expansion of the collection and treatment systems for domestic sewage and more intensified control of the destination of industrial effluents, are essential to prevent environment deterioration of Babitonga Bay. Urban runoff is an important source of diffuse pollution for several contaminants such as heavy metals (mainly Cu, Pb and Cd) and organic compounds, which can contaminate the receiving water bodies (Prestes et al., 2006), and should be considered in the practices of mitigation and control of impacts on coastal environments.

## Conclusions

This study assessed the impacts of multiple stressors on the structure of macrobenthic communities in urbanized estuarine gradients. Despite the interaction and possible magnification effects of the different local stressors, our multivariate models allowed us to identify gradients and effects on the fauna not previously studied. The many distinct anthropogenic pressures do interact within estuaries, combining effects inside these naturally “stressed” environments (Elliott & Quintino, 2007) and generating responses which are difficult to distinguish (Ellis et al., 2015).

Our results indicate that the majority of Babitonga Bay present a non-contaminated to moderately contaminated status and special attention should be given to the inner areas. Despite some inconsistencies in the ecological classification provided by AMBI, our results suggest that the environmental quality pointed out by this index was in general satisfactory for the studied gradients. The metal gradient showed the weakest correlation to AMBI, a possible reflection of transference of the logics and theories of responses to organic enrichment to metals. In such cases, the ecological classification of taxa by the index should be evaluated under the perspective of the action of inorganic genotoxic contaminants. Our results can support future management practices and policies for metal and organic contamination in Brazilian and eventually along South American coastal waters that do not yet benefit from parameters involving the benthic fauna as a robust bioindicator of anthropic impacts.

## Supplemental Information

10.7717/peerj.12427/supp-1 Supplemental Information 1 Average recoveries and standard deviation (*n* = 3) of certified values for trace metals. Standard reference material (BCR701), Recoveries (% Rec), Method Detection Limits (MDL) and Method Quantification Limits (MQL).

10.7717/peerj.12427/supp-2 Supplemental Information 2 Distance-based multivariate multiple regression analysis (DISTLM) of the relationships between macrofauna and hydrodynamic gradient. Selection criterion: AICc. Selection procedure: Step-wise. DF: 33.

10.7717/peerj.12427/supp-3 Supplemental Information 3 Distance-based multivariate multiple regression analysis (DISTLM) of the relationships between macrofauna and metal contamination gradient. Selection criterion: AICc. Selection procedure: Step-wise. DF: 33.

10.7717/peerj.12427/supp-4 Supplemental Information 4 Distance-based multivariate multiple regression analysis (DISTLM) of the relationships between macrofauna and organic enrichment gradient. Selection criterion: AICc. Selection procedure: Step-wise. DF: 33.

10.7717/peerj.12427/supp-5 Supplemental Information 5 DistLM between stressor variables and macrofauna showing the percentage explained in benthic community composition (R^2^) by each of the three groups, each pairwise combination and all three groups.

10.7717/peerj.12427/supp-6 Supplemental Information 6 Number of occurrences of each class of Geoaccumulation Index (*Igeo*) and Contamination Factor (*CF*) for Cr, Cu, Ni and Zn at the 35 sampling sites in Babitonga Bay.

10.7717/peerj.12427/supp-7 Supplemental Information 7 Abiotic sediment variables for each sampling site.

10.7717/peerj.12427/supp-8 Supplemental Information 8 Mean abundance for each species at the sampling sites.

## Additional Information and Declarations

### Field Study Permissions

The following information was supplied relating to field study approvals (*i.e*., approving body and any reference numbers): The collection of fauna was approved by the Brazilian Ministry of Environment (ICMBIO) (42056-1).

### Data Availability

The following information was supplied regarding data availability: The raw measurements are available in the Supplementary Files.
