# Supplementary material for: Macrobenthic community responses to multiple environmental stressors in a subtropical estuary
Source: PeerJ. 2021 Dec 7;9:e12427. doi: 10.7717/peerj.12427 (PMC8663631; doi:10.7717/peerj.12427)
Supplement: Supplemental Information 1 — Standard reference material (BCR701), Recoveries (% Rec), Method Detection Limits (MDL) and Method Quantification Limits (MQL). [file peerj-09-12427-s001.docx]

| Element | BCR701 (mg.Kg^-1^) | Present  Study (mg.Kg^-1^) | % Rec | | MDL (mg.L^-1^) | | MQL (mg.L^-1^) | |
| --- | --- | --- | --- | --- | --- | --- | --- | --- |
| Cr | 272 | 301,29 ± 4,43 | 110,77 | 0,0013 | | 0,0046 | |  |
| Cu | 275 | 321,49 ± 6,24 | 116,91 | 0,0026 | | 0,0092 | |  |
| Ni | 103 | 106,46 ± 1,67 | 103,36 | 0,0024 | | 0,0087 | |  |
| Zn | 454 | 513,65 ± 5,31 | 113,14 | 0,0053 | | 0,0189 | |  |
