# Supplementary material for: Macrobenthic community responses to multiple environmental stressors in a subtropical estuary
Source: PeerJ. 2021 Dec 7;9:e12427. doi: 10.7717/peerj.12427 (PMC8663631; doi:10.7717/peerj.12427)
Supplement: Supplemental Information 2 — Selection criterion: AICc. Selection procedure: Step-wise. DF: 33. [file peerj-09-12427-s002.docx]

| **MARGINAL TESTS** | | | | | | | | |
| --- | --- | --- | --- | --- | --- | --- | --- | --- |
|  | Variable | SS(trace) | Pseudo-F | P | Prop. |  |  |  |
| 1 | *Magelona papillicornis* | 1,89 | 1,39 | 0,244 | 0,040 |  |  |  |
| 2 | *Caprella sp.* | 6,09 | 4,92 | 0,034 | 0,130 |  |  |  |
| 3 | *Polydora sp.* | 15,44 | 16,20 | 0,001 | 0,329 |  |  |  |
| 4 | *Sternaspis sp.* | 4,30 | 3,33 | 0,076 | 0,092 |  |  |  |
| 5 | *Bulla striata* | 12,10 | 11,47 | 0,003 | 0,258 |  |  |  |
| 6 | *Streblospio benedicti* | 8,54 | 7,35 | 0,013 | 0,182 |  |  |  |
| 7 | Capitellidae | 10,53 | 9,55 | 0,004 | 0,224 |  |  |  |
| 8 | *Glycinde multidens* | 11,64 | 10,89 | 0,003 | 0,248 |  |  |  |
| 9 | *Prionospio steenstrupi* | 9,80 | 8,72 | 0,006 | 0,209 |  |  |  |
| 10 | *Owenia sp.* | 0,15 | 0,11 | 0,749 | 0,003 |  |  |  |
| 11 | *Hermundura tricuspis* | 5,11 | 4,04 | 0,054 | 0,109 |  |  |  |
| 12 | *Sigambra sp.* | 14,19 | 14,31 | 0,001 | 0,302 |  |  |  |
| 13 | Nemertea | 12,06 | 11,42 | 0,002 | 0,257 |  |  |  |
| 14 | *Isolda pulchella* | 0,28 | 0,20 | 0,670 | 0,006 |  |  |  |
| 15 | *Heleobia australis* | 0,22 | 0,15 | 0,712 | 0,005 |  |  |  |
| 16 | *Scoloplos sp.* | 1,31 | 0,95 | 0,330 | 0,028 |  |  |  |
| 17 | *Sthenelais limicola* | 3,37 | 2,55 | 0,121 | 0,072 |  |  |  |
| 18 | *Aricidea sp.* | 0,06 | 0,04 | 0,842 | 0,001 |  |  |  |
| 19 | *Sphenia fragilis* | 0,09 | 0,06 | 0,808 | 0,002 |  |  |  |
| 20 | Syllidae | 1,33 | 0,96 | 0,341 | 0,028 |  |  |  |
| 21 | Mysida | 1,43 | 1,04 | 0,283 | 0,031 |  |  |  |
| 22 | Sabellidae | 0,04 | 0,03 | 0,877 | 0,001 |  |  |  |
| 23 | Brachyura | 0,01 | 0,01 | 0,939 | 0,000 |  |  |  |
| 24 | *Magelona variolamellata* | 0,00 | 0,00 | 0,988 | 0,000 |  |  |  |
| 25 | Lumbrineridae | 0,22 | 0,16 | 0,697 | 0,005 |  |  |  |
| 26 | *Neanthes bruaca* | 1,28 | 0,93 | 0,341 | 0,027 |  |  |  |
| **SEQUENTIAL TESTS** | | | | | | | | |
|  | Variable | AICc | SS(trace) | Pseudo-F | P | Prop. | Cumul. | res.df |
|  | +Polydora sp. | 0,65 | 15,44 | 16,20 | 0,001 | 0,329 | 0,329 | 33 |
|  | +Nemertea | -2,07 | 4,28 | 5,04 | 0,032 | 0,091 | 0,421 | 32 |
|  | +Mysida | -5,50 | 4,27 | 5,78 | 0,028 | 0,091 | 0,512 | 31 |
|  | +Capitellidae | -7,51 | 2,91 | 4,36 | 0,045 | 0,062 | 0,574 | 30 |
|  | *+Bulla.striata* | -8,61 | 2,17 | 3,53 | 0,071 | 0,046 | 0,620 | 29 |
|  | *-Polydora sp.* | -9,01 | 1,33 | 2,17 | 0,150 | 0,028 | 0,591 | 30 |
|  | +Syllidae | -11,08 | 2,55 | 4,45 | 0,042 | 0,054 | 0,646 | 29 |
| **BEST SOLUTION** | | | | | | | | |
|  | AICc | R^2^ | RSS | No.Vars | Selections | | | |
|  | -11,08 | 0,646 | 16,62 | 5 | 5;7;13;20;21 | | | |
