# Supplementary material for: Macrobenthic community responses to multiple environmental stressors in a subtropical estuary
Source: PeerJ. 2021 Dec 7;9:e12427. doi: 10.7717/peerj.12427 (PMC8663631; doi:10.7717/peerj.12427)
Supplement: Supplemental Information 3 — Selection criterion: AICc. Selection procedure: Step-wise. DF: 33. [file peerj-09-12427-s003.docx]

| **MARGINAL TESTS** | | | | | | | | |
| --- | --- | --- | --- | --- | --- | --- | --- | --- |
|  | Variable | SS(trace) | Pseudo-F | P | Prop. |  |  |  |
| 1 | *Magelona papillicornis* | 0,02 | 0,00 | 0,951 | 0,000 |  |  |  |
| 2 | *Caprella sp.* | 0,51 | 0,14 | 0,686 | 0,004 |  |  |  |
| 3 | *Polydora sp.* | 7,28 | 2,12 | 0,156 | 0,060 |  |  |  |
| 4 | *Sternaspis sp.* | 2,96 | 0,83 | 0,351 | 0,025 |  |  |  |
| 5 | *Bulla striata* | 2,57 | 0,72 | 0,394 | 0,021 |  |  |  |
| 6 | *Streblospio benedicti* | 29,78 | 10,82 | 0,004 | 0,247 |  |  |  |
| 7 | Capitellidae | 0,22 | 0,06 | 0,808 | 0,002 |  |  |  |
| 8 | *Glycinde multidens* | 3,41 | 0,96 | 0,331 | 0,028 |  |  |  |
| 9 | *Prionospio steenstrupi* | 12,51 | 3,82 | 0,061 | 0,104 |  |  |  |
| 10 | *Owenia sp.* | 3,12 | 0,88 | 0,333 | 0,026 |  |  |  |
| 11 | *Hermundura tricuspis* | 1,71 | 0,47 | 0,485 | 0,014 |  |  |  |
| 12 | *Sigambra sp.* | 38,33 | 15,37 | 0,001 | 0,318 |  |  |  |
| 13 | Nemertea | 0,48 | 0,13 | 0,724 | 0,004 |  |  |  |
| 14 | *Isolda pulchella* | 8,61 | 2,54 | 0,121 | 0,071 |  |  |  |
| 15 | *Heleobia australis* | 16,19 | 5,11 | 0,033 | 0,134 |  |  |  |
| 16 | *Scoloplos sp.* | 10,37 | 3,10 | 0,086 | 0,086 |  |  |  |
| 17 | *Sthenelais limicola* | 10,24 | 3,06 | 0,089 | 0,085 |  |  |  |
| 18 | *Aricidea sp.* | 13,75 | 4,24 | 0,053 | 0,114 |  |  |  |
| 19 | *Sphenia fragilis* | 0,00 | 0,00 | 0,996 | 0,000 |  |  |  |
| 20 | Syllidae | 3,72 | 1,05 | 0,315 | 0,031 |  |  |  |
| 21 | Mysida | 10,11 | 3,02 | 0,109 | 0,084 |  |  |  |
| 22 | Sabellidae | 0,36 | 0,10 | 0,718 | 0,003 |  |  |  |
| 23 | Brachyura | 2,84 | 0,79 | 0,371 | 0,023 |  |  |  |
| 24 | *Magelona variolamellata* | 0,13 | 0,04 | 0,856 | 0,001 |  |  |  |
| 25 | Lumbrineridae | 29,91 | 10,88 | 0,003 | 0,248 |  |  |  |
| 26 | *Neanthes bruaca* | 1,28 | 0,35 | 0,561 | 0,011 |  |  |  |
| **SEQUENTIAL TESTS** | | | | | | | | |
|  | Variable | AICc | SS(trace) | Pseudo-F | P | Prop. | Cumul. | res.df |
|  | +Sigambra | 34,31 | 38,33 | 15,37 | 0,001 | 0,318 | 0,318 | 33 |
|  | +Lumbrineridae | 25,58 | 22,43 | 11,98 | 0,001 | 0,186 | 0,504 | 32 |
|  | +Heleobia.australis | 20,92 | 11,16 | 7,10 | 0,015 | 0,092 | 0,596 | 31 |
|  | +Mysida | 20,64 | 4,03 | 2,71 | 0,114 | 0,033 | 0,629 | 30 |
|  | +Scoloplos | 20,43 | 3,83 | 2,72 | 0,109 | 0,032 | 0,661 | 29 |
|  | +Magelona.papillicornis | 20,42 | 3,53 | 2,64 | 0,120 | 0,029 | 0,690 | 28 |
| **BEST SOLUTION** | | | | | | | | |
|  |  | AICc | R^2^ | RSS | No.Vars | Selections | | |
|  |  | 20,42 | 0,690 | 37,35 | 6 | 1;12;15;16;21;25 | | |
