# Supplementary material for: Macrobenthic community responses to multiple environmental stressors in a subtropical estuary
Source: PeerJ. 2021 Dec 7;9:e12427. doi: 10.7717/peerj.12427 (PMC8663631; doi:10.7717/peerj.12427)
Supplement: Supplemental Information 4 — Selection criterion: AICc. Selection procedure: Step-wise. DF: 33. [file peerj-09-12427-s004.docx]

| **MARGINAL TESTS** | | | | | | | | |
| --- | --- | --- | --- | --- | --- | --- | --- | --- |
|  | Variable | SS(trace) | Pseudo-F | P | Prop. |  |  |  |
| 1 | *Magelona papillicornis* | 35,00 | 3,24 | 0,078 | 0,089 |  |  |  |
| 2 | *Caprella sp.* | 4,57 | 0,39 | 0,563 | 0,012 |  |  |  |
| 3 | *Polydora sp.* | 15,58 | 1,37 | 0,255 | 0,040 |  |  |  |
| 4 | *Sternaspis sp.* | 0,65 | 0,06 | 0,821 | 0,002 |  |  |  |
| 5 | *Bulla striata* | 0,04 | 0,00 | 0,960 | 0,000 |  |  |  |
| 6 | *Streblospio benedicti* | 52,20 | 5,07 | 0,030 | 0,133 |  |  |  |
| 7 | Capitellidae | 4,94 | 0,42 | 0,515 | 0,013 |  |  |  |
| 8 | *Glycinde multidens* | 0,79 | 0,07 | 0,801 | 0,002 |  |  |  |
| 9 | *Prionospio steenstrupi* | 14,52 | 1,27 | 0,276 | 0,037 |  |  |  |
| 10 | *Owenia sp.* | 20,47 | 1,82 | 0,187 | 0,052 |  |  |  |
| 11 | *Hermundura tricuspis* | 10,49 | 0,91 | 0,354 | 0,027 |  |  |  |
| 12 | *Sigambra sp.* | 69,55 | 7,12 | 0,010 | 0,178 |  |  |  |
| 13 | Nemertea | 0,10 | 0,01 | 0,928 | 0,000 |  |  |  |
| 14 | *Isolda pulchella* | 26,85 | 2,43 | 0,121 | 0,069 |  |  |  |
| 15 | *Heleobia australis* | 3,62 | 0,31 | 0,600 | 0,009 |  |  |  |
| 16 | *Scoloplos sp.* | 52,98 | 5,16 | 0,029 | 0,135 |  |  |  |
| 17 | *Sthenelais limicola* | 20,48 | 1,82 | 0,181 | 0,052 |  |  |  |
| 18 | *Aricidea sp.* | 26,86 | 2,43 | 0,123 | 0,069 |  |  |  |
| 19 | *Sphenia fragilis* | 25,28 | 2,28 | 0,136 | 0,065 |  |  |  |
| 20 | Syllidae | 4,13 | 0,35 | 0,565 | 0,011 |  |  |  |
| 21 | Mysida | 81,07 | 8,61 | 0,006 | 0,207 |  |  |  |
| 22 | Sabellidae | 4,61 | 0,39 | 0,554 | 0,012 |  |  |  |
| 23 | Brachyura | 9,49 | 0,82 | 0,380 | 0,024 |  |  |  |
| 24 | *Magelona variolamellata* | 4,69 | 0,40 | 0,532 | 0,012 |  |  |  |
| 25 | Lumbrineridae | 47,81 | 4,59 | 0,035 | 0,122 |  |  |  |
| 26 | *Neanthes bruaca* | 9,00 | 0,78 | 0,381 | 0,023 |  |  |  |
| **SEQUENTIAL TESTS** | | | | | | | | |
|  | Variable | AICc | SS(trace) | Pseudo-F | P | Prop. | Cumul. | res.df |
|  | +Mysida | 80,81 | 81,07 | 8,61 | 0,006 | 0,207 | 0,207 | 33 |
|  | *+Scoloplos sp.* | 76,34 | 55,35 | 6,93 | 0,015 | 0,141 | 0,348 | 32 |
|  | *+Sigambra sp.* | 71,24 | 50,22 | 7,59 | 0,008 | 0,128 | 0,476 | 31 |
| **BEST SOLUTION** | | | | | | | | |
|  |  | AICc | R^2^ | RSS | No.Vars | Selections | | |
|  |  | 71,24 | 0,476 | 205,21 | 3 | 12;16;21 | | |
