# Supplementary material for: Macrobenthic community responses to multiple environmental stressors in a subtropical estuary
Source: PeerJ. 2021 Dec 7;9:e12427. doi: 10.7717/peerj.12427 (PMC8663631; doi:10.7717/peerj.12427)
Supplement: Supplemental Information 5 [file peerj-09-12427-s005.docx]

| **Groups** | | | | | | | |
| --- | --- | --- | --- | --- | --- | --- | --- |
| 1 | Hydrodynamic |  |  |  |  |  |  |
| 2 | Organic Enrichment |  |  |  |  |  |  |
| 3 | Metals |  |  |  |  |  |  |
|  |  |  |  |  |  |  |  |
| **Marginal Tests**: Hydrodynamic gradient included. | | | | | | | |
|  | Group | SS(trace) | Pseudo-F | P | Prop. | res.df | regr.df |
| 1 | Hydrodynamic | 19446 | 4,78 | 0,000 | 0,230 | 32 | 3 |
| **Specified Solution** | | | | | | | |
|  | Adj R^2 | R^2^ | RSS | No.Groups | Selections |  |  |
|  | 0,182 | 0,230 | 65085 | 1 | 1 |  |  |
|  |  |  |  |  |  |  |  |
| **Marginal Tests**: Organic enrichment gradient included. | | | | | | | |
|  | Group | SS(trace) | Pseudo-F | P | Prop. | res.df | regr.df |
| 2 | Organic Enrichment | 6430 | 2,72 | 0,001 | 0,076 | 33 | 2 |
| **Specified Solution** | | | | | | | |
|  | Adj R^2 | R^2^ | RSS | No.Groups | Selections |  |  |
|  | 0,048 | 0,076 | 78101 | 1 | 2 |  |  |
|  |  |  |  |  |  |  |  |
| **Marginal Tests**: Metal contamination gradient included. | | | | | | | |
|  | Group | SS(trace) | Pseudo-F | P | Prop. | res.df | regr.df |
| 3 | Metals | 20434 | 2,39 | 0,000 | 0,242 | 30 | 5 |
| **Specified Solution** | | | | | | | |
|  | Adj R^2 | R^2^ | RSS | No.Groups | Selections |  |  |
|  | 0,141 | 0,242 | 64097 | 1 | 3 |  |  |
|  |  |  |  |  |  |  |  |
| **Marginal Tests**: Hydrodynamic and organic enrichment gradients included. | | | | | | | |
|  | Group | SS(trace) | Pseudo-F | P | Prop. | res.df | regr.df |
| 1 | Hydrodynamic | 19446 | 4,78 | 0,000 | 0,230 | 32 | 3 |
| 2 | Organic Enrichment | 6430 | 2,72 | 0,001 | 0,076 | 33 | 2 |
| **Specified Solution** | | | | | | | |
|  | Adj R^2 | R^2^ | RSS | No.Groups | Selections |  |  |
|  | 0,221 | 0,289 | 60075 | 2 | 1;2 |  |  |
|  |  |  |  |  |  |  |  |
| **Marginal Tests**: Hydrodynamic and metal contamination gradients included. | | | | | | | |
|  | Group | SS(trace) | Pseudo-F | P | Prop. | res.df | regr.df |
| 1 | Hydrodynamic | 19446 | 4,78 | 0,000 | 0,230 | 32 | 3 |
| 3 | Metals | 20434 | 2,39 | 0,000 | 0,242 | 30 | 5 |
| **Specified Solution** | | | | | | | |
|  | Adj R^2 | R^2^ | RSS | No.Groups | Selections |  |  |
|  | 0,251 | 0,384 | 52111 | 2 | 1;3 |  |  |
|  |  |  |  |  |  |  |  |
| **Marginal Tests**: Organic enrichment and metal contamination gradients included. | | | | | | | |
|  | Group | SS(trace) | Pseudo-F | P | Prop. | res.df | regr.df |
| 2 | Organic Enrichment | 6430 | 2,72 | 0,001 | 0,076 | 33 | 2 |
| 3 | Metals | 20434 | 2,39 | 0,000 | 0,242 | 30 | 5 |
| **Specified Solution** | | | | | | | |
|  | Adj R^2 | R^2^ | RSS | No.Groups | Selections |  |  |
|  | 0,160 | 0,283 | 60573 | 2 | 2;3 |  |  |
|  |  |  |  |  |  |  |  |
| **Marginal Tests**: Hydrodynamic, organic enrichment and metal contamination gradients included. | | | | | | | |
|  | Group | SS(trace) | Pseudo-F | P | Prop. | res.df | regr.df |
| 1 | Hydrodynamic | 19446 | 4,78 | 0,000 | 0,230 | 32 | 3 |
| 2 | Organic Enrichment | 6430 | 2,72 | 0,001 | 0,076 | 33 | 2 |
| 3 | Metals | 20434 | 2,39 | 0,000 | 0,242 | 30 | 5 |
| **Specified Solution** | | | | | | | |
|  | Adj R^2 | R^2^ | RSS | No.Groups | Selections |  |  |
|  | 0,287 | 0,433 | 47893 | 3 | All |  |  |
