# Supplementary material for: Macrobenthic community responses to multiple environmental stressors in a subtropical estuary
Source: PeerJ. 2021 Dec 7;9:e12427. doi: 10.7717/peerj.12427 (PMC8663631; doi:10.7717/peerj.12427)
Supplement: Supplemental Information 6 [file peerj-09-12427-s006.docx]

|  |  |  | **Occurrences** | | | |
| --- | --- | --- | --- | --- | --- | --- |
| ***Igeo*** | **Class** | **Sediment Quality** | **Cr** | **Cu** | **Ni** | **Zn** |
| <0 | 0 | Absence of contamination | 15 | 35 | 27 | 8 |
| 0-1 | 1 | Absent to moderately contaminated | 20 | - | 8 | 25 |
| 1-2 | 2 | Moderately contaminated | - | - | - | 2 |
| 2-3 | 3 | Moderate to heavily contaminated | - | - | - | - |
| 3-4 | 4 | Heavily contaminated | - | - | - | - |
| 4-5 | 5 | Heavily to extremely contaminated | - | - | - | - |
| >5 | 6 | Extremely contaminated | - | - | - | - |
| ***CF*** |  | **Contamination degree** | **Cr** | **Cu** | **Ni** | **Zn** |
| <1 |  | Low | 5 | 32 | 7 | 2 |
| 1-3 |  | Moderate | 30 | 3 | 28 | 31 |
| 3-6 |  | Considerable | - | - | - | 2 |
| >6 |  | Very High | - | - | - | - |
